# Caries prevention in permanent teeth - basic recommendations of the German S 3 guideline

**DOI:** 10.1007/s00784-026-06880-1

**Published:** 2026-04-18

**Authors:** Nadine Schlueter, Elmar Hellwig, Werner Geurtsen, Stefan Rupf

**Affiliations:** 1 Department of Conservative Dentistry, Periodontology and Preventive Dentistry, Hannover Medical School, Carl-Neuberg-Str. 1, 30625 Hannover, Germany; 2 Department of Operative Dentistry and Periodontology, Medical Center, Faculty of Medicine, University of Freiburg, Hugstetterstr. 55, 79106 Freiburg, Germany; 3 Synoptic Dentistry, Saarland University, Kirrberger Str. 100, Bldg. 73, 66421 Homburg, Germany

**Keywords:** Nutrition advice, fluoride, oral hygiene, prevention program, chlorhexidine, saliva

## Abstract

**Objectives:**

Caries is still one of the most common diseases in Germany and world-wide, and most adults, children and adolescents are affected during their lifetime. The primary goal of oral disease prevention is therefore maintaining healthy teeth that are either free from decay or sufficiently restored without any secondary caries.

**Materials and methods:**

This updated S3-guideline is intended to provide scientifically sound information on basic measures and recommendations for caries prevention in the permanent dentition, based on the current state of knowledge on etiology and pathogenesis of caries. This guideline applies to all persons with permanent dentition, provides information for dentists, educators, teachers, parents and other multipliers. Its core messages can be divided into measures to be taken by the population at home, and measures recommended and carried out by staff members in dental practices.

**Results:**

The classic caries prevention pillars (tooth brushing, fluoride use, nutritional advice and regular dental consultations) have been expanded to seven recommendations: (I) Oral hygiene, including interdental cleaning; (II) Risk-adapted recommendations for fluoride use as part of daily oral hygiene, plus use of fluoridated table salt; (III) Reducing sugar consumption. In addition, in dental practices (IV) the participation in risk-adapted and structured prevention programs should be recommended, (V) application of further highly concentrated fluoride preparations such as varnishes, (VI) application of chlorhexidine in case of orthodontic treatment or exposed root surfaces, and (VII) fissure sealing in areas at high risk of caries development should be considered.

**Conclusions:**

These measures can help promote lifelong dental health.

**Clinical relevance:**

The recommendations of the guideline should be implemented in individual and professional caries prevention.

**Supplementary Information:**

The online version contains supplementary material available at 10.1007/s00784-026-06880-1.

## Introduction

Dental caries is a localized disease process of teeth caused by the interaction of an azidogenic, microbial dysbiotic biofilm and certain food components, in particular low-molecular carbohydrates. The dysbiosis of the microbial biofilm develops dynamically and is influenced by microbial metabolic processes and interactions of the various microbial species with each other and with the host. Both the composition and the “dynamics” of the oral biofilm over time vary greatly from individual to individual, with corresponding consequences for the prevention and treatment of biofilm-associated diseases [1]. The transition from “healthy” to “diseased” is decisively determined by host factors and biofilm dynamics. If an imbalance in biofilm composition and activity develops, the dental hard tissue can be demineralized (“ecological plaque hypothesis”) [2].

Caries occurs when the microbial acid effect on the tooth surface exceeds the protective and remineralizing influences, thus causing demineralization. The development of caries is mainly due to behavioral attitudes. With suitable preventive measures, carious lesions can be avoided (primary prevention) or their progression can be stopped or remineralized (secondary prevention) [3].

Caries prevention is one of the main goals in dentistry. Individualized preventive dental care strategies are of particular importance, as changes in individual life circumstances can directly influence personal dietary or oral hygiene habits and intrinsic factors such as saliva quantity and composition. Caries is one of the most common diseases worldwide. In Germany, its prevalence is still around 93% in adults and 22% in 12-year-olds and caries is still responsible for around 50% of tooth loss [4].

Caries should be understood as a disease process. Delaying invasive interventions results in a better tooth survival, avoids a destructive and costly restoration cycle and, in addition, results in an increase in quality of life over the individuals’ entire lifespan. Therefore, the prevention of tooth decay, which is subject matter of the present guideline, is of paramount importance.

The guideline presented here provides reliable information on basic measures and recommendations for caries prevention in permanent dentition. The recommendations are based on the current scientific knowledge of the etiology and pathogenesis of caries. The target patient group of the guideline are persons with permanent teeth.

## Materials and methods

The methodology for preparing this guideline was based on the Association of the Scientific Medical Societies in Germany (AWMF) guidelines [5]. A systematic literature search was carried out for the period 2015 to 2022, extended to 2023 during the consensus process using the NCBI PubMed database as well as a hand search in Google Scholar and pertinent German journals. The results of the previous version (2002 to 2014) of this guideline were also included. Seven key questions (PICO questions, for description of PICO(S) aspects see Suppl. Table 1) were formulated, from which recommendations should be derived. The search strategy included selected search terms (Suppl. Table 2) that were determined on the basis of the defined PICO questions:

1. Which oral hygiene strategies for reducing dental biofilm at home are capable of reducing caries increment?
2. What chemical intervention on the biofilm leads to a reduction in caries increment?
3. Does the use of prevention programs lead to a reduction in caries increment?
4. Which fluoridation measures lead to a reduction in caries increment?
5. What influence does diet, specifically the intake of low-molecular carbohydrates, have on caries increment?
6. Can the stimulation of salivary flow lead to a reduction of caries increment in the permanent dentition?
7. How do fissure sealants influence caries increment?

Number of studies found per PICO question can be found in Suppl. Table 3. The available evidence of studies was assessed according to the Scottish Intercollegiate Guidelines Network (SIGN) [6] (see Suppl. Table 4).

Based on the results of the systematic literature search and the evaluation of the literature evidence, the recommendations of the guideline were drawn up in accordance with the specifications of the AWMF. In a first step, all recommendations were evaluated by thirteen mandate holders of participating scientific associations (see Suppl. Table 5) in a Delphi process. Afterwards, modified recommendations were finally discussed and agreed at consensus conferences (for recommendation grading scheme see Suppl. Table 6a and for classification of consensus strength see Suppl. Table 6b) under neutral moderation by the AWMF resulting in a total of 28 recommendations and statements (21 evidence-based recommendations, six consensus-based recommendations and one statement). The final version was agreed by all accountable bodies on 28 January 2025; following, the guideline is valid until 27 January 2030 [7]. In the event of significant changes to the facts, e.g. new relevant evidence, an adjustment, if necessary, an amendment or update of the guideline, including a possible change to the recommendations will be done.

### Target groups

The sector addressed is outpatient care with therapeutic and/or preventive primary dental care. The target user group and addressees of the guideline are dentists of all specialties, dental assistants and dental hygienists as well as nutritionists. It also provides information for educators, teachers, parents and other multipliers.

No special recommendations for pre-school children and for groups with a particularly high caries risk, such as radiotherapy patients or people with severely restricted mobility as well as persons with certain disabilities (e.g. intellectual disability, cerebral palsy, autism spectrum disorder) are given. The guideline does not cover (micro-) invasive caries therapy or the prevention of non-caries-caused dental hard tissue loss such as erosive or abrasive tooth wear. Dietary recommendations relate exclusively to caries prevention. Furthermore, this guideline does not contain any statements on the prevention of periodontal diseases (please see the respective guidelines; Suppl. Table 7).

### Consensual and agreed opinions and recommendations

All recommendations can be found in Table 1, along with information on whether they are consensus- or evidence-based, the strength of the consensus, the recommendation level according to the AWMF, and the level of evidence in the literature, in the case of evidence-based recommendations, according to the SIGN. In case of lack of evidence, consensus based recommendations have been formulated and the respective literature, if available, is mentioned.

**Table 1:** Display of 21 evidence-based recommendations, six consensus-based recommendations and one statement of the S3 guideline for caries prevention in permanent teeth

No.§	Recommendations	Consensus strength# (Yes/No/Abstain)	Recommen-dation Level*	Evidence level*	Reference
Mechanical processes to reduce the biofilm					
1E	Teeth **shall** be cleaned at least twice a day with a toothpaste containing fluoride.	Strong (13/0/0)	A	1++ 1++	[8, 9]
2E	The minimum brushing time **should** not be less than two minutes.	Strong (13/0/0)	B	1- 2++ 2+	[10] [11] [12]
3E	An individually suitable systematic **should** be taught so that all tooth surfaces are reached equally during oral hygiene and the biofilm is reduced or removed equally on all surfaces.	Strong (13/0/0)	B	1+ 2+	[13] [14]
1 C	To date, no tooth brushing technique has been identified that is superior to any other. An individually suitable technique **shall** be recommended for oral hygiene instructions. Regular checks **shall** be made to ensure that the chosen oral hygiene technique is not used traumatically.	Strong (11/0/2)	---	---	[15–17]
2 C	Electric toothbrushes **can be** recommended to improve biofilm reduction. Attention **shall** be paid to correct use, including individual systematics	Strong (12/0/1)	---	---	[18–20]
3 C	It is biologically plausible that teeth **should** be brushed after meals from a cariological point of view.	Strong (14/0/0)	---	---	Expert consensus
4E	It is biologically plausible that regular interdental cleaning has a caries-protective effect. It **should** therefore be carried out regularly several times a week.	Strong (14/0/0)	B	1++ 2+ 2+ 2+	[21] [22] [23] [24]
Chemical influence on the biofilm					
5E	During orthodontic treatment with fixed appliances or in the exposed root area, the professional application of CHX varnishes containing at least 1% CHX **can be** recommended to prevent caries.	Strong (13/0/1)	0	1++ 2++ 2+	[25, 26] [27]
Prevention programs					
6E	Children and adolescents **shall** be offered a needs- and risk-oriented structured prevention program for caries prevention.	Strong (14/0/0)	A	1++	[28]
7E	Motivation and instruction for caries prevention **should** be lifelong.	Strong (14/0/1)	B	1++ 1+ 1+ 1- 2+	[28] [29] [30] [31] [32]
Fluoridation measures					
8E	The use of fluoride-containing toothpaste with at least 1’000 ppm fluoride is a broadly effective and efficient caries prevention measure. For this reason, teeth **shall** be brushed with a toothpaste containing at least 1’000 ppm fluoride from the time point permanent teeth erupt. The caries-preventive effectiveness increases with the increase in tooth brushing frequency from once to twice a day and with increasing fluoride concentration in the toothpaste. (For the use of fluoride-containing toothpaste in primary dentition, the authors of the guideline refer to the guideline Fluoridation measures for caries prevention in children and adolescents, AWMF register no. 083 − 001)	Strong (14/0/0)	A	1++ 1+	[33] [34]
9E	To prevent root surface caries, a more concentrated toothpaste (5’000 ppm fluoride) **shall** be used permanently. Their use **can be** considered for patients undergoing orthodontic treatment with fixed appliances.	Strong (11/0/2)	A 0	1++ 1++	[35] [36]
10E	Children and adolescents **shall** have a fluoride varnish applied at least twice a year. The local application of fluoride varnish **can be** carried out independently of any broad-spectrum fluoridation measures that have already been carried out. In children and adolescents with a greatly increased risk of caries, the frequency of fluoride application **shall** be more than twice (usually four times a year), as an improved caries-reducing effect can then be expected.	Strong (10/0/0)	A 0 A	1+ 1+	[37] [34]
11E	The use of fluoride-containing varnishes **shall** also be considered as an additional caries prophylactic measure in adults, especially if there is an increased risk of root surface caries.	Strong (11/0/2)	A	1++ 1++ 1++ 2++ 2+	[38] [39] [35] [40] [41]
12E	In children and adolescents, a fluoride gel **can be** applied weekly (individually by brushing) or 2–4 times a year (professionally in the dental practice). Local fluoride application **can be** carried out independently of any broad-spectrum fluoridation measures that have already been carried out.	Strong (12/0/0)	0	1++ 2++	[42] [43]
13E	The use of fluoride-containing gels **shall** also be considered as an additional caries prophylactic measure in adults, particularly if there is an increased risk of root surface caries.	Strong (10/0/3)	A	2++	[44]
4 C	The use of fluoridated table salt **shall** be recommended as a basic measure to prevent tooth decay, depending on the fluoride concentration in drinking water.	Strong (12/0/1)	---	---	[34] [45]
14E	Since the effect of fluoride-containing rinsing solutions is independent of the use of other fluoride-containing preparations such as toothpastes, the use of a fluoride-containing mouth rinse **shall** be recommended for children and adolescents (from the age of 6 years) with an increased risk of caries (e.g. fixed orthodontic treatment).	Consensus (10/1/2)	A	1++	[46]
15E	In adults, fluoride-containing mouth rinses **should** be recommended for caries prevention, especially for the prevention of root surface caries.	Strong (11/0/2)	B	1++ 1++	[46] [47]
Dietary advices					
16E	It **shall** be recommended that the proportion of free sugars in the total energy intake should not exceed 10%.	Strong (10/0/0)	A	1++	[48] (for < 10% free sugars)
17E	The recommendation **shall** be made to reduce the proportion of free sugars in the total energy intake to less than 5% in individual cases.	Strong (10/0/0)	A	1+ 1+	[48] (for < 5% free sugars) [49]
5 C	Frequent consumption of sugary foods and/or drinks at mealtimes is associated with the development of tooth decay. The recommendation **shall** be made to reduce the consumption of products containing free sugars in particular.	Strong (10/0/0)	---	---	[50–54]
18E	The recommendation to avoid sugary snacks **shall** be given.	Strong (10/0/0)	A	2- 2-	[52, 54] [53]
19E	The recommendation to avoid sugar intake before bedtime after oral hygiene **shall** be given.	Strong (11/0/0)	A	1- 2+	[55] [56]
20E	There is a linear relationship between the intake of drinks with added sugar and the development of caries. The recommendation to avoid drinks with added sugar **shall** be given.	Strong (11/0/0)	A	1++ 1-	[57] [58]
1 S	Unprocessed starch has low cariogenicity.	Consensus (8/1/3)	---	1- 2++	[59] [60]
21E	It **shall** be recommended to avoid the consumption of foods with processed starch, also in combination with sugars, as this increases the risk of caries.	Strong (11/0/1)	A	1- 2++	[59] [60]
Saliva stimulation					
6 C	Regularly chewing of sugar-free gum can help preventing tooth decay and, therefore, chewing **can be** recommended particularly after meals.	Strong (11/0/0)	---	---	[61] [62]

“§ “E”: evidence-based recommendation, “C”: consensus-based recommendation, “S”: statement without recommendation; * Only relevant for evidence-based recommendations; # variations in number of persons voted depend on number of participants in various consensus meetings (for definition of consensus strength according to AWMF guidelines [5], see supplement Table 6b). Evidence levels were given according to SIGN [6] and recommendation level was given according to the AWMF guidelines [5] for evidence based recommendations (A: strong recommendation, shall or shall not, B: recommendation, should or should not, 0: recommendation open, can be considered or can be dispensed; for further details please see supplement Table 6a).“

## Results

1. **Mechanical processes to reduce the dental biofilm**

The term oral hygiene measures encompasses all strategies that serve to reduce, eliminate or prevent the formation of biofilm with pathogenic potential from or on all tooth surfaces facing the oral cavity. Caries is caused by the metabolic activity of the pathogenic bacterial biofilm on the teeth. It therefore seems biologically plausible that caries can be prevented by adequately influencing this biofilm. This can be achieved mechanically by reducing or eliminating the biofilm, or chemically by applying specific active substances.

The mechanical procedures to reduce biofilm include measures to clean the smooth and occlusal surfaces as well as the interdental spaces. It can be deduced from the pathomechanism of caries that a reduction in biofilm on the tooth surface results in a reduction in the incidence of caries. However, biofilm reduction is often merely a surrogate parameter that is used as a measure of possible caries reduction due to its plausibility. To underline this, various studies have shown that there is a correlation between the amount of biofilm on the tooth surface and the occurrence of caries [63–65]. The quality and extent (quantity) of biofilm removal is crucial. Both parameters can depend on the frequency of mechanical cleaning of the teeth, the duration of the cleaning process, the systematic and technique used and the individual execution of the cleaning efforts.

It is regularly stated that the recording of brushing success in the context of studies says little about habitual oral hygiene, as participation in a study alone leads to an improvement in cleaning performance of up to 20% (Hawthorne effect). This is basically correct, but brushing performance is quite constant within an individual [66]. It can therefore be assumed that the Hawthorne effect has only minor effect as it is most likely that areas generally neglected or habitually not cleaned are also not cleaned in case of study participation. Studies that only consider the surrogate parameter of biofilm reduction can therefore still be considered to have a certain significance, as it can be assumed that areas identified as biofilm-covered in these studies represent areas with a high risk of caries.

The caries-protective effect of fluorides is undisputed (see 6. Fluoridation Measures, e.g. [8, 33, 34]). Therefore, oral hygiene measures are nowadays usually carried out with fluoride-containing preparations. As a result, the effect of mechanical biofilm removal can no longer be separated from the effect of fluoride [9]. There are no adequately conducted clinical studies from earlier years, before fluoride was used regularly, that prove that caries can be prevented by mechanical oral hygiene measures alone. Only a few recent studies consider fluoride as a separate variable in their evaluations. Overall, these studies present inconsistent results, are often based on self-reports and were frequently conducted on pre-school children. These studies showed that the type of toothpaste used had no significant influence on the study results, but the frequency of mechanical biofilm removal did so [67–69]. Further studies showed a significant influence of both parameters [70, 71], while others found a correlation with oral hygiene quality, but not with fluoride application [72]. Therefore, it is not possible to clearly deduce what part mechanical removal of the biofilm or fluoride application plays in the caries-preventive effect of the oral hygiene measure. Due to the clear fluoride effect and the basic recommendation for the caries-preventive use of fluorides, it will no longer be feasible to conduct prospective randomized clinical studies that can clarify the effect of the respective component in isolation for ethical reasons in the future.

### Tooth brushing frequency

Systematic reviews have shown a correlation between brushing frequency and caries incidence. Brushing twice or more times a day has a higher caries-preventive effect than mechanical biofilm removal by brushing only once a day [9] or less frequently [8].

Regarding the surrogate criterion biofilm reduction some studies clearly demonstrated a link between tooth brushing and reduction in biofilm index values, however, it remains unclear whether a reduction in biofilm to the measured extent (minus 30–53%) is associated with a reduction in caries risk [10].

### Brushing duration and brushing systematics

An increase in reduction of biofilm reduction was measured with increasing brushing duration either from 30 s to 3 min (increase by 55%) or from 1 min to 2 min (increase in plaque index value reduction from 27% to 41%) [10]. Extending the brushing time beyond this did not lead to further biofilm reduction [11] .

Observational studies showed that around 10–20% of test subjects included did not reach the palatal surfaces in the upper jaw or lingual surfaces in the lower jaw or only insufficiently [14]. Insufficiently means that, if dividing the jaws into sextants, the lingual and palatal surfaces were not sufficiently reached during toothbrushing and in less than six sextants. Therefore, only an instruction in a suitable systematic represents an option for improving brushing efficiency with the goal to reach all areas [66]. Interestingly, even if it is plausible that a suitable tooth brushing systematic leads to an improved biofilm reduction due to reaching all surfaces, there is still lack in final evidence for effectiveness [13]. Only few clinical studies are available on the influence of brushing frequency and duration on caries development. These few studies have demonstrated an effect for permanent molars in schoolchildren [12].

### Tooth brushing technique

Observational studies have shown that mostly circular or horizontal movements are performed during tooth brushing [14]. Even if specific tooth brushing techniques are recommended for biofilm removal, reviews have shown that no tooth brushing technique is superior to another [13, 15, 16]. Consequently, no specific technique can be recommended with regard to biofilm reduction. However, attention should be paid to the non-traumatic application of oral hygiene techniques. Brushing with a toothbrush alone without toothpaste does not lead to mechanically induced dental hard tissue loss in either enamel or dentine when used as intended. However, the combination of toothpaste and toothbrush can lead to mechanically induced damage, especially in exposed dentin [17]. Gingival abrasions can sometimes be observed when excessive pressure is applied [17].

### Type of toothbrush

Both manual toothbrushes and powered toothbrushes can be used to remove biofilm. Powered toothbrushes remove biofilm more effectively than manual toothbrushes. The average additional percentage reduction in plaque index values with powered toothbrushes was 11% in short-term studies (up to three months) and 21% in long-term studies (longer than three months) [18]. Another meta-analysis found moderate evidence for the superiority of powered toothbrushes over manual toothbrushes [73], while another found high evidence [19]. Regarding the different types of powered toothbrushes, no clear superiority of one type over another can be found as only small difference in biofilm reduction (surrogate parameter) could be found making the clinical relevance for caries reduction questionable [19, 20].

### Brushing time point

It is biologically plausible that teeth should be brushed after meals in order to remove food debris that could be available as a substrate for cariogenic germs. However, there is no evidence for these recommendations from randomized clinical studies. The long-standing recommendation to postpone toothbrushing after an acidic meal, can no longer be upheld on the basis of the available studies [74–76].

### Interdental hygiene and devices for interdental hygiene

Around 30–40% of the tooth surfaces are located in the interdental space. Toothbrushes cannot fully penetrate the interdental space, therefore aids for cleaning interdental spaces are required. These aids are dental floss or interdental brushes, as well as interdental sticks and powered aids, including oral irrigators.

A systematic review from 2019 on the use of interdental hygiene aids at home in addition to tooth brushing examined their influence on the development of periodontal disease and caries [21]. A total of 35 randomized clinical trials were included. None of the studies examined caries as an endpoint, so the surrogate parameter biofilm reduction was used as a measure. Only very low evidence was found that the use of dental floss in addition to toothbrushing reduces more biofilm than toothbrushing alone (study duration 3–6 months), little evidence was found for the additional use of interdental brushes, wooden sticks have no additional benefit in terms of biofilm reduction (study duration three months).

Overall, there are very few studies with caries as an endpoint. A study on schoolchildren showed a lower incidence of caries for both primary teeth and the first permanent molars. However, no distinction was made between the localization of caries [22]). Self-reported use of dental floss of adults was able to significantly reduce caries incidence both overall and proximally, however, with no difference between use 1-3x per week and 4-7x per week [23]. Comparable results were shown for seniors, although the caries-preventive effects were less pronounced in this age group [24].

Overall, the lack of evidence for a benefit of dental floss or interdental brushes or sticks should not be equated with evidence for a lack of benefit; their use is biologically plausible, and the recommendation to use them should not be neglected as their use may also have a positive effect on other oral diseases [77].

2. **Chemical influence on the biofilm**

In addition to mechanical aids, various chemical compounds are used in toothpastes and rinsing solutions, gels or varnishes to influence metabolism and prevent growth of cariogenic microorganisms.

Reviews show that the use of such preparations leads to a reduction in bacteria. However, the data on a general caries-reducing effect is weak and contradictory [40, 78]. In particular, no additional caries-preventive effect can be observed with the use of chemical plaque inhibitors in patients who take adequate caries prevention with fluoride preparations. Some older reviews showed a caries-reducing effect for chlorhexidine varnishes in fissures of erupting molars and in root caries [25, 79].

### Chlorhexidine (CHX)

A systematic Cochrane review investigated the caries-preventive effect of oral hygiene products containing chlorhexidine (including toothpastes, mouthwashes, varnishes, gels, chewing gum and sprays) on children and adults [26]. Only randomized controlled studies in which a placebo or no treatment was used, or in which different carrier substances were compared, were considered. Studies in which chlorhexidine was combined with fluoride in one preparation, or where chlorhexidine was compared with fluoride applications, were excluded. The review revealed that there was limited evidence to suggest that chlorhexidine is more effective than a placebo or no treatment in preventing caries in children and adolescents.

Figuero et al. (2017) were able to show in a systematic review that mechanical plaque control, combined with the use of fluoride-containing preparations, can contribute to a reduction in caries. However, the use of CHX solutions does not appear to be suitable for additional caries reduction [80].

CHX varnish is also used to prevent caries during orthodontic treatment. Two systematic reviews with meta-analysis concluded that the application of a CHX varnish during orthodontic treatment with fixed appliances has caries-protective and -preventive effects [81]. However, it should be noted that the study populations of the selected studies were due to fixed orthodontic appliances high-risk patients and that only a small number of studies were included. Comparable was found in a study in split mouth design using a 1% CHX-1% thymol varnish compared to a placebo varnish in orthodontic patients on a monthly base [27]. After six months, the number of newly formed, initial caries lesions was lower in the CHX-thymol treated teeth. Another indication for the use of CHX varnish is the prevention of root caries. In a systematic review, Wierichs and Meyer-Lückel (2015) concluded that CHX varnishes can be effectively used to reduce progression and prevent initiation of root caries [39].

### Other chemical compounds

In a systematic review, Slayton et al. (2018) concluded that no recommendation can be made for the use of other chemical compounds in the context of arresting or reversing non-cavitated carious lesions [3]. No studies showing superiority of these compounds exist.

A systematic review with subsequent meta-analysis concluded that a combination of local fluoridation with an antibacterial agent (povidone iodide, chlorhexidine, xylitol, triclosan, cetylpyridinium chloride) shows better caries-preventive efficacy than fluoride application alone [37]. However, this review pooled different combinations of preparations, different age groups and different examination methods. When determining the certainty of the evidence, it became apparent that it is very heterogeneous overall. If the study is broken down according to the different antibacterial agents that were used together with fluoridation measures, it can be seen that, with the exception of products containing xylitol, no additional positive effect was found with regard to the caries-reducing effect of the combination preparations in comparison to the fluoride preparations.

3. **Prevention programs**

With an overall concept that includes the use of different prophylactic measures, it is possible to significantly reduce the development of carious defects [82]. Reliable data is available, particularly for adolescents. These were summarized in a systematic review with meta-analysis [28]. It was shown that multifactorial programs for adolescents are superior to interventions that only provide information.

Prevention programs often include a combination of the factors information, motivation and instruction at different intervals as well as various forms of fluoride application. The effect of different programs on plaque and gingivitis in young adults were compared. All programs resulted in a reduction in plaque and gingival indices. Most of the programs, which included fluoridation measures, resulted in a caries reduction of 30–70%. It has not yet been shown that a particular combination of measures or certain fluoride preparations were more effective than others [31]. Groups with an increased risk of caries benefit in particular from prevention programs. The quality of information and motivation play a decisive role [30]. Adults can also benefit from prevention programs. A retrospective analysis of data from people aged 15–80 years taken part in an oral health program for adults showed that participation resulted in significantly less interdental plaque and a lower tendency bleeding on probing after just one session than before participation. People with poorer initial values benefited more than people who already had better plaque and bleeding values from the outset. The prevalence and incidence of caries was associated with higher interdental plaque values in this analysis [32].

In a randomized clinical study, the influence of monthly professional dental cleanings and individual oral hygiene instruction by dental hygienists compared with individual oral hygiene in 146 Swedish nursing home residents was investigated. Over a period of six months, a 17% reduction in active root caries lesions was achieved in the intervention group compared to 4% in the control group [29].

4. **Fluoridation measures**

One of the most important cornerstones of individual and group caries prevention is the use of various fluoride-containing preparations. Numerous meta-analyses and systematic reviews [8, 33, 34, 38, 83, 84] concluded that the use of fluoride-containing preparations leads to different but significant caries reduction rates. In principle, the total fluoride intake from swallowing the corresponding preparations and the fluoride intake from food or drinks should not exceed a value of 0.05–0.07 mg fluoride/kg body weight per day [85, 86]. When recommending fluoridation measures, the fluoride content in drinking water must be considered.

### Toothpaste containing fluoride

Various reviews have shown that the daily use of fluoride-containing toothpaste (1000 to 1500 ppm fluoride) is an effective method for caries prevention in permanent teeth in children and adolescents [8, 33, 34, 87]. They clearly have shown that the effectiveness in children and adolescents is dose-dependent and that there is also a dependence on the frequency of use. A cariostatic effect of fluoride-containing toothpaste can also be demonstrated in adults. In addition, the use of a highly concentrated fluoride toothpaste (5000 ppm F^-^) appears to play an important role in the prevention of root surface caries [88, 89]. This was confirmed in two systematic reviews with meta-analysis [35, 39]. Another systematic Cochrane Review [36] found that the use of this highly concentrated toothpaste reduces the development of incipient caries lesions in patients with fixed orthodontic appliances more effectively than the use of a conventional fluoride toothpaste. However, this statement is based on the results of only one study.

### Fluoride varnish

Several systematic reviews, some with meta-analysis, have shown that fluoride-containing varnishes are effective in preventing caries [34, 37–40, 90–93]. A recent single study was also able to show that a fluoride varnish application in newly erupted molars has a similar caries-reducing effect as a fissure sealant [94]. These findings are in concordance with the EAPD guideline on fluoridation measures [34], which also states that the application of a fluoride varnish can be used effectively to prevent caries. In patients with a risk of root surface caries, treatment with fluoride varnish every three months leads to a reduction in caries with a high level of evidence [40, 41, 47, 95].

### Fluoride gel

In a systematic review with meta-analysis was concluded that there is moderate evidence for a clear caries-preventive effect for highly concentrated fluoride gels (28% compared to placebo and 38% compared to no fluoride application) [42]. This effect is independent of other fluoridation measures or the type of e.g. gel application (professional with applicator or brushing at home) [43, 96], resulting in a recommendation of the use of highly concentrated fluoride gel from the age of 6 years in the EAPD fluoride guideline [34]. Such fluoride gels can also be recommended for the prevention and arrest of root surface caries [44]. The application of fluoride gels (mainly in Germany, gels with a fluoride concentration of 1.25% are used) leads to inactivation or remineralization of initial caries lesions, especially in caries-active patients. However, the results are very heterogeneous.

### Table salt containing fluoride

A community-based fluoridation measure that can be considered is the use of fluoride-containing table salt. However, the scientific evidence for the cariostatic efficacy of fluoride-containing table salt is weak for countries where other fluoridation measures (fluoride toothpaste, fluoride varnish etc.) are used. Jordan et al. (2017) were able to show in a more recent clinical study that table salt containing fluoride has a caries-preventive effect if no other significant fluoridation measures are available [45]. In line with the recommendations of the EAPD fluoride guideline, fluoridated table salt can be considered as part of a community-based prevention program [34] taking all fluoride sources and the maximum for total daily intake of fluoride into account. Other sources for fluoride in nutrition, such as fluoride added to drinking water or milk, are not allowed in Germany and has therefore not been part of the guideline and consensus process.

### Rinsing solutions containing fluoride

The use of fluoride-containing mouth rinses has a caries-preventive effect. A systematic review in the Cochrane Library (Marinho et al., 2016) found that the regular supervised use of fluoride-containing rinses in children and adolescents (230 or 900 ppm F^-^) leads to a caries reduction of 27% (DMFS) or 23% (DMFT) [46]. Based on the individual studies included in the review, most of which were published in the 1970s and 1980s, the caries-reducing effect appears to be independent of caries activity and the use of other fluoride-containing preparations. The EAPD fluoride guideline confirms this statement [34]. In children and adolescents with an increased risk of caries, the daily supervised use of mouth rinses (at a concentration of 0.05% NaF) or the weekly supervised use of a mouth rinsing solution (0.2% NaF) leads to a significant reduction in the increase in caries. From a toxicological point of view, fluoride-containing rinsing solutions should only be used from persons who can rinse and spit out. This is for children mostly from the age of 6 years. Restrictions, however, exists not only for very young children but also for persons with certain disabilities (e.g. cerebral palsy, severe intellectual disability). Two systematic reviews confirm that the use of fluoride-containing rinsing solutions also contributes to caries prevention in adults (especially on root surfaces) [46, 95].

### Fluoride tablets

Fluoride tablets do not play a significant role in caries prevention in the permanent dentition. There are no recent studies that show sufficient evidence for the use of fluoride tablets in the prevention of caries in permanent teeth. Only for children up to an age of 6 years, the recommendation of fluoride tablets might be meaningful in case of very high risk of caries according to the EAPD guideline [34].

5. **Dietary recommendations**

There is convincing evidence from studies in animal models as well as from epidemiological and experimental studies in humans that there is a link between the amount and frequency of free sugar intake and the development of caries. In a meta-analysis of archaeological finds, the authors found an association between tooth loss ante mortem and tooth decay [97]. From Pre-Christian findings over the 9th century up to the 17th century and after the 18th century, there was a clear increase of caries prevalence from around 6% to 18% and of ante mortem tooth loss from 9% − 11% up to 26%. The authors attribute this to the increasing availability of sugar. Until around 1970, a strong positive correlation between sugar consumption and caries prevalence was found in many countries [98]. After the introduction of fluoridation measures, however, only a weak correlation can be shown [99].

### Low molecular weight carbohydrates (sugar)

All mono- and disaccharides are considered as sugars. Free sugars are all sugars added to food by manufacturers or consumers, as well as sugars that are naturally present in e.g. honey, fruit juices, syrups. Although other fermentable carbohydrates are also cariogenic, epidemiological studies show that starchy staple foods and fresh fruit are only weakly associated with caries. A systematic review examined the relationship between the amount of sugar consumed (per day or year or as a percentage of total energy intake (%E)) and the extent of caries [48] and found evidence for most studies (17/23 studies) that limiting the intake of free sugars to < 10%E, in some studies < 5%E reduces the risk of tooth decay. With an estimated total energy intake of 2000 kcal per day, the recommendation of < 10%E corresponds to a maximum intake of 50 g of free sugars [100]. In a meta-analysis and a simulation taking existing clinical studies into account, the authors observed a relative reduction in DMFT in 15-year-olds of 13% when food contained 20% less sugar [101]. A linear relationship between sugar intake and caries development was found in another systematic review, leading to the suggestion by the authors to further reduce sugar intake to 2–3% of energy intake [49]. One study found a link between sugar intake and tooth decay in adults aged 30–89 years [50]. An association study demonstrated a link between high sugar consumption in children aged 3 years and increased rates of caries between the ages of 3 and 16 years [51]. Older studies showed that frequent sugary snacks increase the caries risk [52–54]. The timing of sugar intake is also important. Schoolchildren included into a cohort study consuming sugar before bedtime have a 2.4-fold increased risk of developing cavitated caries lesions [56]. However, in a systematic review only weak evidence on this question was found [55]. The results suggest that limiting the consumption of free sugars before and at bedtime could reduce the risk of tooth decay. A current review is available on the influence of sweetened beverages on caries development [57]. This study included 38 cross-sectional studies, 26 of which were classified as high quality. A dose-response gradient with high evidence was observed for caries. In contrast, no link was found between the intake of 100% fruit juice and tooth decay [58].

The cariogenicity of uncooked starch is very low and that of highly processed and heated starch is higher, but not as high as that of sugars [102, 103]. A systematic review of studies from 1963 to 2011 concluded that there is no association between starch consumption and tooth decay [59]. However, for the combination of sugars and starch another systematic review concluded that frequent consumption of sugary and starchy foods between meals increased the risk of tooth decay [60]. A systematic review showed that a higher BMI is associated with a higher DMFT [104].

### Vegetarian diet

In a systematic review on the influence of a vegetarian diet, a higher risk of erosions and a lower DMFT value were found. However, the quality of the available studies was low. A meta-analysis, which only included studies with adults, showed no influence of a vegetarian diet on DMFT. The association between a vegetarian diet and caries is inconsistent [105].

### Sugar substitutes

Sugar substitutes (polyols) such as sorbitol, xylitol and erythritol and sweeteners such as cyclamate and aspartame cannot or only to a very small extent be metabolized by oral microorganisms to acids, and are therefore not cariogenic [106, 107]. Although the scientific evidence is not clear, it is biologically plausible that the risk of caries can be reduced by completely or partially replacing sugars with sugar substitutes or sweeteners [108]. A comparison of the use of xylitol, sorbitol and erythritol as sweets, which were given to schoolchildren over three years, resulted for erythritol in a slight reduction in caries growth for a further three years of observation. However, caries incidence could not be prevented in any of the intervention groups (xylitol, sorbitol, erythritol) [109]. In a randomized and blinded study on schoolchildren with low caries activity, no influence of xylitol sweeteners on caries growth was found [110]. In four reviews [107, 111–113], a possible caries-preventive effect of xylitol was examined independently of its use as xylitol chewing gum. All four reviews concluded that there is insufficient evidence for a caries-preventive effect of xylitol. Therefore, no recommendation was given.

### Probiotics

Initial data are available on the use of probiotics, especially *Lactobacillus rhamnosus* and *L. reuteri*, to prevent caries in children, but no recommendations can yet be made for their use in permanent dentition [114].

6. **Saliva stimulation**

The development and progression of caries are influenced by protective salivary factors. Of particular importance is the neutralization of acids by the buffer systems of saliva, the cleansing of the oral cavity from food debris (clearance) and the remineralizing effect of saliva. Sufficient saliva (0.5–1 L/day) is therefore considered a cornerstone of oral health and it seems biologically plausible that chewing sugar-free gum induces increase in saliva flow and salivary pH, which can reduce the incidence and progression of tooth decay [61]. A recent systematic review and meta-analysis revealed that chewing sugar-free gum, especially those containing xylitol as a sugar substitute, significantly reduces caries incidence [62]. In a systematic review, based on six clinical studies, good to very good evidence was found that sugar-free chewing gums can have an anti-cariogenic effect due to saliva stimulation, especially after meals [115]. Other systematic reviews also concluded that regular chewing of sugar-free gum has a caries-preventive effect and can therefore be recommended as part of the basic measures for caries prevention [107, 116]. However, it should be noted that excessive gum chewing could exacerbate temporomandibular joint disorders in patients [117].

7. **Fissure sealants**

Fissures and pits of erupting or just erupted molars are considered high-risk caries predilection sites in children and adolescents. In particular if they exhibit a complex or deep fissure relief. As recently a new German S3 guideline on fissure sealing was published, this preventive measure was not subject of the present guideline, even if it is part of the preventive concept in Germany. With regard to fissure sealing, please refer to the basic recommendations of the S3 guideline “Fissure and pit sealing” (AWMF 083 − 002; https://register.awmf.org/de/leitlinien/detail/083-002) [118]. (For a complete list of links to other guidelines see Suppl. Table 7)

## Conclusion

Effective caries prevention is not based on single interventions, but on the coordinated interaction of seven key preventive measures (Table 2), which can be divided into measures to be taken by the population at home (recommendations 1, 4, 5 and 6), and measures recommended and carried out by qualified staff members in dental practices (recommendations 2, 3, 4 and 7). Measures at home include the elimination or control of microbial factors (dysbiotic biofilm) on the dental hard tissues, motivating people to eat a diet that promotes dental health (avoidance of sugar) and use fluoridated salt, the promotion of the protective mechanisms of saliva (chewing of sugar free chewing gums after meals), and the use of various fluoridation measures to counteract the demineralization of the dental hard tissues and to promote the remineralization of incipient signs of demineralization. Measures to be done in the dental practice include the recommendation to participate in risk-adapted and structured prevention programs, the application of further highly concentrated fluoride preparations such as varnishes, the application of chlorhexidine in case of orthodontic treatment or exposed root surfaces, and fissure sealing of fissures with high risk of developing caries. These measures can help ensure lifelong dental health.

**Table 2:** Summary of basic recommendations of the S3 guideline on caries prevention in permanent teeth

Measure	Recommendations
1. Mechanical processes to reduce the biofilm:	As a basic prevention, patients shall brush their teeth at least twice a day with a fluoride-containing toothpaste in such a way that a clinically effective reduction or complete removal of the biofilm results. Different toothbrushes can be used depending on the patients’ preference and abilities. As food residues and biofilm cannot be sufficiently removed from the interdental space with tooth brushing alone, devices for proximal space hygiene (dental floss, interdental brushes) should also be used.
2. Chemical biofilm control	During orthodontic treatment with fixed appliances or in the exposed root area, the professional application of CHX varnishes containing at least 1% CHX can be recommended to prevent caries.
3. Prevention programs	Caries can be significantly reduced by combining various prophylactic measures. In particular, patients with an increased risk of caries shall be recommended to participate in structured prevention programs.
4. Fluoridation measures	Patients shall brush their teeth with a fluoride-containing toothpaste. In addition, fluoride-containing table salt shall always be used in the household. In addition, the use of toothpastes with an increased fluoride concentration or fluoride-containing varnishes, gels or rinsing solutions may be indicated (especially for patients with active caries - risk adapted recommendation).
5. Nutrition	The total amount of daily sugar intake and the number of sugary meals (main meals and snacks), including sugary drinks, shall be kept as low as possible. Foods and drinks without added sugar shall be preferred.
6. Saliva stimulation	Regularly chewing sugar-free gum can also help prevent tooth decay and chewing can therefore be recommended, particularly after meals.
7. Fissure sealants	Fissures and pits at risk of caries should be sealed as part of a prevention concept.

## Supplementary Information

Below is the link to the electronic supplementary material.

Supplementary Material 1

## Data Availability

No datasets were generated or analysed during the current study.
