# Correction to: Caries prevention in permanent teeth - basic recommendations of the German S 3 guideline

**DOI:** 10.1007/s00784-026-06988-4

**Published:** 2026-06-26

**Authors:** Nadine Schlueter, Elmar Hellwig, Werner Geurtsen, Stefan Rupf

**Affiliations:** 1 Department of Conservative Dentistry, Periodontology and Preventive Dentistry, Hannover Medical School, CarlNeuberg-Str. 1, 30625 Hannover, Germany; 2 Department of Operative Dentistry and Periodontology, Medical Center, Faculty of Medicine, University of Freiburg, Hugstetterstr. 55, 79106 Freiburg, Germany; 3 Synoptic Dentistry, Saarland University, Kirrberger Str. 100, Bldg. 73, 66421 Homburg, Germany

**Correction to: Clin Oral Invest**

10.1007/s00784-026-06880-1

In the published paper, the given names and surnames of all the authors were interchanged.

The correct order of names and affiliations is as follows:

Nadine Schlueter^1^ · Elmar Hellwig^2^ · Werner Geurtsen^1^ · Stefan Rupf^3^

The original article has been corrected.

