# Supplementary material for: Caries prevention in permanent teeth - basic recommendations of the German S 3 guideline
Source: Clin Oral Investig. 2026 Apr 18;30(5):182. doi: 10.1007/s00784-026-06880-1 (PMC13091887; doi:10.1007/s00784-026-06880-1)
Supplement: Supplementary file 1 — Supplementary Material 1 [file 784_2026_6880_MOESM1_ESM.docx]

**Suppl. Table 1:** Description of PICO(S) aspects

**PICO-question 1: Mechanical processes to reduce the dental biofilm**

| **PICO(S) ASPECT** | **DESCRIPTION** |
| --- | --- |
| **POPULATION** | Persons with permanent dentition and permanent teeth in mixed dentition  Exceptions are:   - Children without permanent teeth - Groups with a particularly high risk of caries, such as patients who have undergone radiotherapy - Persons with severely limited mobility - Dental malformations |
| **PROBLEM / DISEASE** | Measures for prophylaxis/primary prevention of caries Exceptions:   - Invasive measures for caries treatment (secondary prophylaxis measures) - Measures for the prevention of non-caries- related loss of hard tooth substance (e.g. dental erosion and abrasion) - Measures for the prophylaxis of periodontal diseases |
| **INTERVENTION** | Mechanical plaque reduction   1. Toothbrushes 2. Interdental hygiene products |
| **COMPARATIVE INTERVENTION** | - |
| **OUTCOMES** | Caries incidence Caries prevalence  (DMF-T/DMF-S, ICDAS) |
| **STUDY DESIGN** | Literature basis   1. Prepared literature: guidelines, systematic reviews, HTA   RCT, CCT, cohort studies, cross-sectional studies, population studies, observational studies |
| **RELEVANT PERIOD** | January 2015–February 2022  Follow-up to the period covered by the existing guideline |
| **LANGUAGES** | English, German |
| **DATABASES** | PubMed, manual search: DZZ, oral prophylaxis, SSO |

| **PICO-ASPECT** | **SYNONYME ENGLISH** |
| --- | --- |
| **POPULATION,**  **PROBLEM / DISEASE** | permanent tooth, permanent teeth, permanent dentition, second dentition, secondary dentition, adult dentition, adult tooth, adult teeth, mixed dentition stage |
| **INTERVENTION** | oral hygiene, tooth cleaning, toothbrushing, tooth brushing, toothbrush, manual toothbrush, powered toothbrush, electric toothbrush, interdental cleaning, interdental cleaning device, dental floss, floss, interdental brush, tooth stick, plaque removal, mechanical cleaning of teeth, plaque control, dental biofilm removal, dental biofilm control, mechanical dental biofilm removal |
| **OUTCOME** | Caries, caries incidence, caries prevalence, dental caries, dental decay, tooth decay, DMF, DMF index, DMF-T, DMF-S, ICDAS, ICDAS index |

**PICO-question 2: Chemical influence on the biofilm**

| **PICO(S) ASPECT** | **DESCRIPTION** |
| --- | --- |
| **POPULATION** | Individuals with permanent dentition and permanent teeth in mixed dentition  Exceptions:   - Children without permanent teeth - Groups with a particularly high risk of caries, such as patients who have undergone radiotherapy - Persons with severely limited mobility - Dental malformations |
| **PROBLEM / DISEASE** | Measures for prophylaxis/primary prevention of caries Exceptions:   - Invasive measures for caries treatment (secondary prophylaxis measures) - Measures for the prevention of non-caries-related loss of hard tooth substance (e.g. dental erosion and abrasion) - Measures for the prophylaxis of periodontal diseases |
| **INTERVENTION** | Chemical influence on biofilm |
| **COMPARATIVE INTERVENTION** | - |
| **OUTCOMES** | Caries incidence Caries prevalence  (DMF-T/DMF-S, ICDAS) |
| **STUDY DESIGN** | Literature basis   1. Processed literature: guidelines, systematic reviews, HTA 2. RCT, CCT, cohort studies, cross-sectional studies, population studies, observational studies |
| **RELEVANT PERIOD** | January 2015 – December 2022  Continuation of the period covered by the existing guideline |
| **LANGUAGES** | English, German |
| **DATABASES** | PubMed, manual search: DZZ, oral prophylaxis, SSO |

| **PICO-ASPECT** | **SYNONYME ENGLISH** |
| --- | --- |
| **POPULATION,**  **PROBLEM / DISEASE** | permanent tooth, permanent teeth, permanent dentition, second dentition, secondary dentition, adult dentition, adult tooth, adult teeth, mixed dentition stage |
| **INTERVENTION** | Chlorhexidine mouthwash, Chlorhexidine gel, Chlorhexidine varnish, Chlorhexidine digluconate, Listerine mouthwash, mouth disinfection, chemical plaque control, chemical biofilm control, quaternary ammonium, compounds, Cetylpyridiniumchlorid, Triclosan |
| **OUTCOME** | Caries increment, delta D-(MF)-T, delta D-(MF)-S, delta RCI (Caries, caries incidence, caries prevalence, dental caries, dental decay, tooth decay, DMF, DMF index, DMF-T, DMF-S, ICDAS, ICDAS index) |

**PICO-question 3: Prevention programs**

| **PICO(S) ASPECT** | **DESCRIPTION** |
| --- | --- |
| **POPULATION** | Individuals with permanent dentition and permanent teeth in mixed dentition  Exceptions:   - Children without permanent teeth - Groups with a particularly high risk of caries, such as patients who have undergone radiotherapy - Persons with severely limited mobility - Dental malformations |
| **PROBLEM / DISEASE** | Measures for prophylaxis/primary prevention of caries Exceptions:   - Invasive measures for caries treatment (secondary prophylaxis measures) - Measures for the prevention of non-caries-related loss of hard tooth substance (e.g. dental erosion and abrasion) - Measures for the prevention of periodontal disease |
| **INTERVENTION** | Prophylaxis programmes |
| **COMPARATIVE INTERVENTION** | - |
| **OUTCOMES** | Caries incidence Caries prevalence  (DMF-T/DMF-S, ICDAS) |
| **STUDY DESIGN** | Literature basis   1. Prepared literature: guidelines, systematic reviews, HTA 2. RCT, CCT, cohort studies, cross-sectional studies, population studies, observational studies |
| **RELEVANT PERIOD** | January 2015 – February 2022  Follow-up to the period covered by the existing guideline |
| **LANGUAGES** | English, German |
| **DATABASES** | PubMed, manual search: DZZ, oral prophylaxis, SSO |

| **PICO-ASPECT** | **SYNONYME ENGLISH** |
| --- | --- |
| **POPULATION,**  **PROBLEM / DISEASE** | permanent tooth, permanent teeth, permanent dentition, second dentition, secondary dentition, adult dentition, adult tooth, adult teeth, mixed dentition stage |
| **INTERVENTION** | professional prophylaxis program, professional oral hygiene instruction, plaque control program, plaque control programme, preventive program, preventive programme, education program, education programme, health care program, health care programme, oral health promotion program, oral health promotion programme, oral-health promotion programs, caries prevention program, caries prevention programme, health promotion, patient education, motivational interviewing, oral health knowledge |
| **OUTCOME** | Caries increment, delta D-(MF)-T, delta D-(MF)-S, delta RCI (Caries, caries incidence, caries prevalence, dental caries, dental decay, tooth decay, DMF, DMF index, DMF-T, DMF-S, ICDAS, ICDAS index) |

**PICO-question 4: Fluoridation measures**

| **PICO(S) ASPECT** | **DESCRIPTION** |
| --- | --- |
| **POPULATION** | Individuals with permanent dentition and permanent teeth in mixed dentition  Excluded are:   - Children without permanent teeth - Groups with a particularly high risk of caries, such as patients who have undergone radiotherapy - Persons with severely limited mobility - Dental malformations |
| **PROBLEM / DISEASE** | Measures for the prophylaxis/primary prevention of caries Exceptions:   - Invasive measures for caries treatment (secondary prophylaxis measures) - Measures for the prevention of non-caries-related loss of hard tooth substance (e.g. dental erosion and abrasion) - Measures for the prophylaxis of periodontal diseases |
| **INTERVENTION** | Fluoridation measures:   1. Fluoride-containing toothpaste 2. Fluoride varnish application 3. Fluoride gel application 4. Fluoride-containing table salt 5. Fluoride-containing mouthwashes |
| **COMPARATIVE INTERVENTION** | - |
| **OUTCOMES** | Caries incidence Caries prevalence  (DMF-T/DMF-S, ICDAS) |
| **STUDY DESIGN** | Literature base   1. Prepared literature: guidelines, systematic reviews, HTA 2. RCT, CCT, cohort studies, cross-sectional studies, population studies, observational studies |
| **RELEVANT PERIOD** | January 2015 – December 2022  Continuation of the period covered by the existing guideline |
| **LANGUAGES** | English, German |
| **DATABASES** | PubMed, manual search: DZZ, oral prophylaxis, SSO |

| **PICO-ASPECT** | **SYNONYME ENGLISH** |
| --- | --- |
| **POPULATION,**  **PROBLEM / DISEASE** | permanent tooth, permanent teeth, permanent dentition, second dentition, secondary dentition, adult dentition, adult tooth, adult teeth, mixed dentition stage |
| **INTERVENTION** | Fluoridated dentifrice, Fluoridated toothpaste, Fluoride toothpaste, Fluoride dentifrice, Fluoride varnish, Fluoride lacquer, Fluoride gel, Fluoridated salt, Salt fluoridation, Fluoride mouthrinse |
| **OUTCOME** | Caries increment, delta D-(MF)-T, delta D-(MF)-S, delta RCI (Caries, caries incidence, caries prevalence, dental caries, dental decay, tooth decay, DMF, DMF index, DMF-T, DMF-S, ICDAS, ICDAS index) |

**PICO-question 5: Dietary recommendations**

| **PICO(S) ASPECT** | **DESCRIPTION** |
| --- | --- |
| **POPULATION** | Individuals with permanent dentition and permanent teeth in mixed dentition  Exceptions:   - Children without permanent teeth - Groups with a particularly high risk of caries, such as patients who have undergone radiotherapy - Persons with severely limited mobility - Dental malformations |
| **PROBLEM / DISEASE** | Measures for prophylaxis/primary prevention of caries Exceptions:   - Invasive measures for caries treatment (secondary prophylaxis measures) - Measures for the prevention of non-caries-related loss of hard tooth substance (e.g. dental erosion and abrasion) - Measures for the prophylaxis of periodontal diseases |
| **INTERVENTION** | Nutritional guidance |
| **COMPARATIVE INTERVENTION** | - |
| **OUTCOMES** | Caries incidence Caries prevalence  (DMF-T/DMF-S, ICDAS) |
| **STUDY DESIGN** | Literature basis   1. Processed literature: guidelines, systematic reviews, HTA 2. RCT, CCT, cohort studies, cross-sectional studies, population studies, observational studies |
| **RELEVANT PERIOD** | January 2015 – February 2022  Connection to the period of the existing guideline |
| **LANGUAGES** | English, German |
| **DATABASES** | PubMed, manual search: DZZ, oral prophylaxis, SSO |

| **PICO-ASPECT** | **SYNONYME ENGLISH** |
| --- | --- |
| **POPULATION,**  **PROBLEM / DISEASE** | permanent tooth, permanent teeth, permanent dentition, second dentition, secondary dentition, adult dentition, adult tooth, adult teeth, mixed dentition stage |
| **INTERVENTION** | cariogenic nutrition, diet, dietary carbohydrates, food habits, sugar, sucrose, fructose, glucose,  disaccharides, sweets, confectionary, honey, xylitol, sorbitol, mannitol, maltitol, lycasin, palatinose, snacks, erythritol, sucralose, stevia |
| **OUTCOME** | Caries increment, delta D-(MF)-T, delta D-(MF)-S, delta RCI (Caries, caries incidence, caries prevalence, dental caries, dental decay, tooth decay, DMF, DMF index, |

**PICO-question 6: Saliva stimulation**

| **PICO(S) ASPECT** | **DESCRIPTION** |
| --- | --- |
| **POPULATION** | Persons with permanent dentition and permanent teeth in mixed dentition Exceptions:   - Children without permanent teeth - Groups with a particularly high risk of caries, such as patients who have undergone radiotherapy - Persons with severely limited mobility - Dental malformations |
| **PROBLEM / DISEASE** | Measures for prophylaxis/primary prevention of caries Exceptions:   - Invasive measures for caries treatment (secondary prophylaxis measures) - Measures for the prevention of non-caries-related loss of hard tooth substance (e.g. dental erosion and abrasion) - Measures for the prevention of periodontal disease |
| **INTERVENTION** | Measures to stimulate saliva production |
| **COMPARATIVE INTERVENTION** | - |
| **OUTCOMES** | Caries incidence Caries prevalence  (DMF-T/DMF-S, ICDAS) |
| **STUDY DESIGN** | Literature basis   1. Prepared literature: guidelines, systematic reviews, HTA 2. RCT, CCT, cohort studies, cross-sectional studies, population studies, observational studies |
| **RELEVANT PERIOD** | January 2015 – March 2022  Connection to the period of the existing guideline |
| **LANGUAGES** | English, German |
| **DATABASES** | PubMed, manual search: DZZ, oral prophylaxis, SSO |

| **PICO-ASPECT** | **SYNONYME ENGLISH** |
| --- | --- |
| **POPULATION,**  **PROBLEM / DISEASE** | permanent tooth, permanent teeth, permanent dentition, secondary dentition, dental caries |
| **INTERVENTION** | Saliva, stimulation, salivary stimulation, chewing gum, lozenges, xylitol, sorbitol, mannitol, erythritol |
| **OUTCOME** | Caries, caries incidence, caries prevalence, dental caries, dental decay, tooth decay, DMF, DMF index, DMF-T, DMF-S, ICDAS, ICDAS index |

**Suppl. Table 2:** Search strategy inclusive selected search terms for each PICO question

| **SEARCH PARAMETERS**  **PICO QUESTION 1: MECHANICAL METHODS FOR REDUCING BIOFILM** | ((permanent teeth) OR (permanent tooth) OR (permanent dentition) OR (second dentition) OR (secondary dentition) OR (adult dentition) OR (adult tooth) OR (adult teeth) OR (mixed dentition)) AND ((Oral Hygiene) OR (toothbrushing) OR (tooth brushing) OR (manual toothbrush) OR (powered toothbrush) OR (electric toothbrush) OR (interdental cleaning) OR (interdental cleaning device) OR (dental floss) OR (floss) OR (interdental brush) OR (tooth stick) OR (tooth cleaning) OR (plaque removal) OR (biofilm removal) OR (toothbrush) OR (mechanical cleaning of teeth) OR (plaque control) OR (dental biofilm removal) OR (dental biofilm control) OR (mechanical dental biofilm removal)) AND ((Caries) OR (DMF-S) OR (DMF-T) OR (DMF) OR (DMF index) OR (ICDAS) OR (ICDAS index) OR (dental decay) OR (dental caries)) | | | | |
| --- | --- | --- | --- | --- | --- |
| **SEARCH PARAMETERS**  **PICO QUESTION 2: CHEMICAL INFLUENCE ON THE BIOFILM** | ((permanent tooth) OR (permanent teeth) OR (tooth) OR (teeth) OR (permanent dentition) OR (second dentition) OR (secondary dentition) OR (adult dentition) OR (dentition) OR (adult tooth) OR (adult teeth) OR (mixed dentition)) AND ((chlorhexidine mouthwash) OR (chlorhexidine gel) OR (chlorhexidine varnish) OR (chlorhexidine digluconate) OR (Listerine mouthwash) OR (mouth disinfection) OR (chemical plaque control) OR (chemical biofilm control) OR (quaternary ammonium compounds) OR (cetylpyridinium chloride) OR (triclosan)) AND ((caries increment) OR (delta D-(MF)-T) OR  (delta D-(MF)-S) OR (RCI) OR (root caries) OR (caries) OR (caries incidence) OR (caries prevalence) OR (dental caries) OR (dental decay) OR (tooth decay) OR (DMF) OR (DMF-T index) OR (DMF-T) OR (DMF-S) OR (ICDAS) OR (ICDAS index) | | | | |
| **SEARCH PARAMETERS**  **PICO QUESTION 3: PREVENTION PROGRAMMES** | (professional prophylaxis programme OR professional oral hygiene instruction OR plaque control programme OR plaque control programme OR preventive programme OR preventive programme OR education programme OR education programme OR health care programme OR health care programme OR oral health promotion programme OR oral health promotion programme OR oral health promotion programmes OR caries prevention programme OR caries prevention programme OR health promotion OR patient education OR motivational interviewing OR oral health  knowledge) AND (caries OR dental caries OR DMF OR DMF index OR ICDAS OR ICDAS index, OR dental decay OR tooth decay) | | | | |
| **SEARCH PARAMETERS**  **PICO QUESTION 4: FLUORIDATION MEASURES** | **Toothpaste**  ((permanent teeth) OR (permanent tooth) OR (permanent dentition) OR (second dentition) OR (secondary dentition) OR (adult dentition) OR (adult tooth) OR  (adult teeth) OR (mixed dentition)) AND  ((fluoridated toothpaste) OR  (fluoridated toothpaste) OR (fluoride toothpaste) OR (fluoride dentifrice)) AND  ((caries) OR (caries prevalence) OR (caries | **Varnish**  ((permanent teeth) OR (permanent tooth) OR (permanent dentition) OR (second dentition) OR (secondary dentition) OR (adult dentition) OR (adult tooth) OR  (adult teeth) OR (mixed dentition)) AND  ((fluoride lacquer) OR (fluoride varnish)) AND  ((caries) OR (caries prevalence) OR (caries incidence) OR (DMF-S)  OR (DMF-T) OR (DMF) OR (DMF | **Gel**  ((permanent teeth) OR (permanent tooth) OR (permanent dentition) OR (second dentition) OR (secondary dentition) OR (adult dentition) OR (adult tooth) OR  (adult teeth) OR (mixed dentition)) AND  ((fluoride gel)) AND ((caries) OR (caries prevalence) OR (caries incidence) OR (DMF-S)  OR (DMF-T) OR (DMF) OR (DMF  index) OR (ICDAS) OR (ICDAS | **Salt**  ((permanent teeth) OR (permanent tooth) OR (permanent dentition) OR (second dentition) OR (secondary dentition) OR (adult dentition) OR (adult tooth) OR  (adult teeth) OR (mixed dentition)) AND ((salt fluoridation) OR  fluoridated salt) AND ((caries) OR (caries prevalence) OR (caries incidence) OR (DMF-S)  OR (DMF-T) OR (DMF) OR (DMF  index) OR | **Mouth rinse**  ((permanent teeth) OR (permanent tooth) OR (permanent dentition) OR (second dentition) OR (secondary dentition) OR (adult dentition) OR (adult tooth) OR  (adult teeth) OR (mixed dentition)) AND  ((fluoride mouth rinse OR mouth rinse)) AND ((caries) OR (caries prevalence) OR (caries incidence) OR (DMF-S)  OR (DMF-T) OR (DMF) OR (DMF  index) OR |
| **SEARCH PARAMETERS**  **PICO QUESTION 5: NUTRITION** | (cariogenic OR nutrition OR diet OR dietary carbohydrates OR food habits OR sugar OR sucrose OR fructose OR glucose OR disaccharides OR sweets OR confectionary OR honey OR xylitol OR sorbitol OR mannitol OR maltitol OR lycasin OR palatinose OR snacks OR erythritol OR sucralose OR stevia) AND (caries OR dental caries OR DMF OR DMF index OR ICDAS OR ICDAS index, OR dental decay OR tooth decay) | | | | |
| **SEARCH PARAMETERS**  **PICO QUESTION 6: SALIVARY STIMULATION** | (permanent teeth) OR (permanent tooth) OR (permanent dentition) OR (secondary dentition) OR (saliva) OR (stimulation) OR (chewing gum) OR (dental caries) OR (caries prevention) OR (xylitol) OR (sorbitol) OR mannitol OR (erythritol) OR (DMF-T) OR (DMF) OR (DMF index) OR (ICDAS) OR (ICDAS index) OR ( dental caries) OR (plaque) | | | | |

**Suppl. Table 3:** Number of studies found (PubMed) within the defined searching time (2015-2022) per PICO question (see also Suppl. Table 1)

| **PICO question - issue** | **Type of study** | **Number of studies found**  **(number of included studies after control of abstracts)** |
| --- | --- | --- |
| (1) Oral Hygiene | All studies  Clinical trials  Randomized Clinical Trials  Systematic Reviews  Meta-analyses | 943  95 (12)  74 (6)  30 (6)  14 (5) |
| (2) Chemical Intervention | All studies  Clinical trials  Randomized Clinical Trials  Systematic Reviews  Meta-analyses | 829  457 (2)  407 (0)  166 (5)  78 (3) |
| (3) Prevention Programs | All studies  Clinical trials  Randomized Clinical Trials  Systematic Reviews  Meta-analyses | 2938  178 (10)  153 (7)  149 (4)  59 (1) |
| (4) Fluoridation  A) dentifrices  B) varnishes/lacquers  C) gels  D) salt  E) mouth rinse | All studies  Clinical trials  Randomized Clinical Trials  Systematic Reviews  Meta-analyses  Reviews  Guidelines  All studies  Clinical trials  Randomized Clinical Trials  Systematic Reviews  Meta-analyses  All studies  Clinical trials  Randomized Clinical Trials  Systematic Reviews  Meta-analyses  Reviews  Guidelines  All studies  Clinical trials  Randomized Clinical Trials  Systematic Reviews  Meta-analyses  All studies  Clinical trials  Randomized Clinical Trials  Systematic Reviews  Meta-analyses  Reviews  Guidelines | 174  44 (3)  39 (0)  15 (5)  9 (4)  15 (10)  1 (1)  149  44 (0)  41 (0)  20 (20)  7 (7)  34  10 (0)  12 (1)  5 (3)  5 (1)  1 (1)  1 (1)  89  5 (1)  4 (0)  10 (0)  5 (0)  72  26 (6)  25 (0)  3 (1)  2 (1)  6 (0)  1 (1) |
| (5) Nutrition advices | All studies  Clinical trials  Randomized Clinical Trials  Systematic Reviews  Meta-analyses | 3389  134 (4)  116 (4)  99 (8)  44 (2) |
| (6) Saliva Stimulation | All studies  Clinical trials  Randomized Clinical Trials  Systematic Reviews  Meta-analyses | 89  1 (0)  0 (0)  1 (1)  0 (0) |
| (7) Fissure Sealant | No literature search performed – reference to S3 guideline on fissure sealing | |

**Suppl. Table 4:** Evidence level according to SIGN

| **1++** | High quality meta-analyses, systematic reviews of randomized controlled trials (RCTs), or RCTs with a very low risk of bias. |
| --- | --- |
| **1+** | Well conducted meta-analyses, systematic reviews of RCTs, or RCTs with a low risk of bias |
| **1-** | Meta analyses, systematic reviews of RCTs or RCTs with a high risk of bias |
| **2++** | High quality of systematic reviews of case-control or cohirt studies, high quality case-control studies or cohort studies with a very low risk of confounding or bias and a high probability that the relationship is causal |
| **2+** | Well conducted case-control studies or cohort studies with a low risk of confounding or bias and a moderate probability that the relationship is causal |
| **2-** | Case-control or cohort studies with a high risk of confounding or bias and a significant risk that the relationship is not causal |
| **3** | Non-analytical studies, e.g. case reports or case series. |
| **4** | Expert opinion |

**Suppl. Table 5:** Participating professional societies/organizations with mandated members of the guideline group

| Professional association/organization (alphabetical order) | Abbreviation | Mandate holder/s |
| --- | --- | --- |
| Federal Association of Pediatric Dentists | BUKiZ | Drs. J. Kant |
| Federal Association of Dentists of the Public Health Service (Bundesverband der Zahnärztinnen und Zahnärzte des Öffentlichen Gesundheitsdienstes e.V.) | BZÖG | Dr. P. Petrakakis |
| Federal Dental Association | BZÄK | Prof. Dr. C. Benz |
| German Society for Geriatric Dentistry | DGAZ | Prof. Dr. M. Noack |
| German Society of Dental Hygienists | DGDH | M. Krauß,  S. Fresmann |
| German Society for Endodontology and Dental Traumatology | DGET | Prof. Dr. K. Galler |
| German Society for Nutrition | DGE | Dr. M. Richter |
| German Society for Pediatric Dentistry | DGKiZ | Prof. Dr. U. Schiffner |
| German Society for Periodontology | DG PARO | Prof. Dr. T. Auschill,  Prof. Dr. R. Cosgarea |
| German Society for Prevention in Dentistry | GPZ | PD Dr. Y. Wagner |
| German Society for Preventive Dentistry | DGPZM | Prof. Dr. S. Zimmer |
| German Society for Prosthetic Dentistry and Biomaterials | DGPro | Prof. Dr. H. Stark,  Prof. Dr. B. Wöstmann |
| German Society for Restorative and Regenerative Dentistry | DGR^2^Z | Prof. Dr. T. Tauböck |
| German Society for Conservative Dentistry | DGZ | Prof. Dr. S. Rupf |
| Free Association of German Dentists | FVDZ | PD Dr. T. Wolf |
| Federal Association of Statutory Health Insurance Dentists | KZBV | Dr. R. J. Hussein,  Dr. J. Beck |
| Association of Medical Professions - Department of Dental Assistants | VMF | S. Gabel  M. Schellmann |

To involve patient representatives, the following patient groups were contacted and invited to contribute to this guideline: Federal Working Group of Patient Centers (BAGP), German Working Group of Self-Help Groups at NAKOS (National Contact and Information Center for the Encouragement and Support of Self-Help Groups) (DAG-SHG), German Disability Council (DBR), Federation of German Consumer Organizations (VZBV), Federal self-help association for people with neck, head and face injuries (T.U.L.P.E. e.V.). There were refusals from the DBR and the VZBV; no feedback was received from the other patient groups. The part on “Patient perspective and quality of life" was therefore written without the involvement of patient representatives.

**Suppl. Table 6a:** Recommendation grading scheme

|  | **Recommendation** | **Recommendation against intervention** | **Description** |
| --- | --- | --- | --- |
| **A** | shall/we recommend | shall not/we do not recommend | Strong recommendation |
| **B** | should/we suggest | should not/we do not suggest | Recommendation |
| **0** | can/can be considered | can be dispensed with | Recommendation open |

**Suppl. Table 6b:** AWMF classification of consensus strength

| **Classification of consensus strength** | |
| --- | --- |
| **Strong consensus** | Approval by > 95% of participants |
| **Consensus** | Approval by > 75 to 95% of participants |
| **Majority approval** | Approval by > 50 to 75% of participants |
| **No consensus** | Approval by < 50% of the participants |

**Suppl. Table 7:** Links to other related guidelines

| Classi-fication | Guidline | Register Number | Link |
| --- | --- | --- | --- |
| S2k | Fluoridation measures for caries prevention in children and adolescents | 083- 001 | https://register.awmf.org/de/leitlinien/  detail/083-001#anmeldung |
| S3 | Fissure and pit sealing | 083- 002 | https://register.awmf.org/de/leitlinien/  detail/083-002 |
| S3 | Chemical biofilm management at home in the prevention and treatment of gingivitis | 083- 016 | https://register.awmf.org/de/leitlinien/  detail/083-016 |
| S3 | Mechanical biofilm management at home in the prevention and treatment of gingivitis | 083- 022 | https://register.awmf.org/de/leitlinien/  detail/083-022 |
| S3 | The German implementation of the S3 guideline "Treatment of Stage I-III Periodontitis" of the European Federation of Periodontology (EFP) | 083- 043 | https://register.awmf.org/de/leitlinien/  detail/083-043 |
| Intern. | Guidelines on the use of fluoride for caries prevention in children: an updated EAPD policy document | EAPD | https://pubmed.ncbi.nlm.nih.gov/  31631242 |
